# Challenges for Workplace Risk Assessment in Home Offices—Results from a Qualitative Descriptive Study on Working Life during the First Wave of the COVID-19 Pandemic in Latvia

**DOI:** 10.3390/ijerph182010876

**Published:** 2021-10-16

**Authors:** Linda Matisāne, Linda Paegle, Lāsma Akūlova, Ivars Vanadziņš

**Affiliations:** Institute of Occupational Safety and Environmental Health, Rīga Stradiņš University, Dzirciema 16, LV-1007 Riga, Latvia; Linda.Paegle@rsu.lv (L.P.); Lasma.Akulova@rsu.lv (L.A.); Ivars.Vanadzins@rsu.lv (I.V.)

**Keywords:** telework, distance work, occupational safety, occupational health, workplace risk assessment, risk assessment methods, home assessment, home office

## Abstract

Epidemiological restrictions due to the COVID-19 pandemic have raised legal and practical questions related to the provision of workplace risk assessment in home offices of teleworkers. The objective of this qualitative study was to analyze practical experience of employers and occupational safety and health experts performing workplace risk assessment in Latvia during the first wave of the COVID-19 pandemic. Our findings suggest that employers have not sufficiently implemented their legal obligations related to workplace risk assessment which can result in an increased number of physical and mental health problems of teleworkers in the short term and in the future. Work from home has shown how different working conditions can be for the same type of work (office work); therefore, the promotion of personalized workplace risk assessment should be encouraged. Even if virtual workplace visits using photos and videos are not the traditional way the workplace risk assessment should be done, it is effective; workers who report that their employers assessed their working conditions report fewer health effects. The experience of workers in participation in workplace risk assessment for telework might change the level and role of worker participation in the management of health and safety hazards at work in general.

## 1. Introduction

The COVID-19 pandemic has been a challenge for companies all over the world. Depending on the local situation, different control measures to mitigate the spreading of SARS-CoV-2 virus have been implemented by the governments. They included physical distancing, restrictions on the freedom of movement, closure of non-essential companies and undertakings, lockdown of entire cities in different parts of the world, etc. [1]. These major changes have also influenced the work of many companies, including adaption of the business models and change of work organization. Most of the companies have also explored work from home as an answer to the COVID-19 pandemic [1].

### 1.1. Telework in Latvia

The definition of telework is given in the national Labour Protection Law that transposes requirements of the Council Directive 89/391/EEC of 12 June 1989 [2,3]. According to this law, telework is defined as “the type of work which could be done in the premises of the employer, but on regular basis is done outside these premises using information and communication technologies” [2]. The European Foundation for the Improvement of Living and Working Conditions uses the term specified in the European framework agreement on telework. According to this definition telework is “a form of organizing and/or performing work, using information technology, in the context of an employment contract/relationship, where work that could be performed at the employer’s premises is carried out away from those premises on a regular basis” [4]. The International Labour Organization defines telework or remote work as “the work performed through the use of information communication technology (ICT’s such as smartphones, tablets, laptops and desktop computers) outside the employer’s premises” [5]. A rather similar definition can be found in literature, e.g., “telecommuting is defined organization and/or execution of tasks performed away from the central offices or production facilities of organizations for a certain period in pre-established work schedules. To this end, information and communication technologies are used to establish communication between workers and to respond to the demands of the organization remotely” [6].

In Latvia, during the first wave of the pandemic, full lockdown was not implemented, therefore telework was only strongly advised. Since 12 March 2020, when the first emergency state was announced in Latvia, the percentage of workers working from home during the COVID-19 pandemic has been changing between 8.9% of the total workforce when COVID-19 restrictions were mild and 22.6% when COVID-19 restrictions were rather strict (for details see Table A1 in Appendix A).

### 1.2. Main Workplace Hazards in Distant Workplaces

Already before the COVID-19 pandemic, it has been recognized that management of occupational safety and health conditions is more difficult due to lack of possibilities to monitor and control the teleworking arrangements, especially in the cases when telework is done from home [1]. When an office worker is working in the office of the employer, workplace hazards can be managed based on workplace risk assessment [7,8]. In addition, the employer takes care of infrastructure, purchases equipment and office furniture, arranges the computer workstations, and also invites occupational safety and health experts and/or ergonomists to improve working conditions on-site [7,8]. However, in the home offices teleworkers set up their workstations by themselves, in many cases without external assistance and training, sometimes even on coffee tables, ironing boards, kitchen and dining tables, old desks, and using living room sofas or beds for seating [7,9]. This results in the situation that many teleworkers have suboptimal working conditions. Studies are reporting that 25 to 40% of teleworkers typically have dining or other nonadjustable chairs without armrests, poor ergonomics related to laptop usage, low monitor heights or poor (e.g., hard) desk worksurfaces in their home offices [10,11].

Due to the size of the living area, not all workers can have a dedicated individual (personal) workstation in their homes, therefore, they have to share these workstations with their children who have to attend school remotely or spouses who also telework during the pandemic [9]. As a solution, setting up shift desks or working in a variety of places throughout the day is quite typical [9]. In addition, environmental factors (e.g., lighting, temperature, humidity, air quality, noise, ergonomics, etc.) could be a problem in home offices and reduce job satisfaction, and this situation can be better addressed by the worker only if he/she has sufficient knowledge [12,13]. The problems related to ergonomics in teleworkplaces are deepened by the fact that at the individual level many workers are less likely to receive sufficient training on ergonomics while working at home, therefore in addition to the lack of suitable equipment and furniture, teleworkers also lack knowledge on how to arrange ergonomic workplaces from the available items [14].

### 1.3. Workplace Risk Assessment for Teleworkplaces

The COVID-19 epidemiological restrictions have deepened all of the above-mentioned problems—the transition to telework was rapid, even massive, and in many countries also forced by government restrictions. Traditionally, problems related to the health and safety of workers are identified and addressed through workplace risk assessment [15]. Concerning workplace risk assessment, the Council Directive 89/391/EEC of 12 June 1989 on the introduction of measures to encourage improvements in the safety and health of workers at work (the so-called Framework Directive) states that “the employer shall be in possession of an assessment of the risks to safety and health at work, including those facing groups of workers exposed to particular risks and decide on the protective measures to be taken and, if necessary, the protective equipment to be used” [3]. These legal provisions are well known as the Member States had to enforce the laws, regulations, and administrative provisions necessary to comply with this Directive by 31 December 1992. For Latvia, these general provisions were applicable since 2002 when the new Labour Protection Law came into force [2].

According to the International Labour Organization, a risk assessment is a workplace examination in order to identify workplace hazards and required control measures [15]. However, due to epidemiological reasons, traditional workplace risk assessment and management methods are not applicable as employers are not allowed to send occupational safety and health experts to identify existing workplace hazards in teleworkers’ homes [8,16]. In this situation, employers and researchers have raised an important social and legal issue—who is responsible for the workplace safety and health of teleworkers in their workplace [7]? Even though it may be difficult for employers to carry out this legal obligation on workplace risk assessment in workers’ homes in the COVID-19 context, in fact, according to the legal acts, employers have the same health and safety responsibilities for home-based teleworkers as for any other workers [1,17].

In general, when discussing methods and approaches used for workplace risk assessment in telework, it is explained that the principles should be the same as for any other workplace [18]. However, the COVID-19 pandemic has required employers and occupational safety and health experts to look for other methods which are not based on physical workplace visits—virtual site-checks with photos or videos and self-addressed checklists are filled in by workers [1,19]. Other employers (with the support of the occupational practitioners) have provided teleconsultations for employees where they listen to their complaints and give advice on how to optimize working conditions despite the pandemic circumstances that take place [20].

### 1.4. Awareness and Knowledge of Teleworkers on Ergonomics

Results from the literature review also suggest that teleworkers report a lower level of awareness and knowledge about ergonomics and safety issues [21]. It can be due to the fact that telework has been identified as a barrier to the implementation of participatory ergonomics programs designed to promote the health, safety, and well-being of workers [14]. Therefore, employers should provide sufficient information and training on health and safety issues for teleworkers covering such topics as ergonomics and criteria for structuring home offices, working in isolation as well as general fire and electrical safety issues. They should also offer ergonomics and safety training or resources to change work habits and improve the physical home-based work environment [14,19,21]. The distribution of a checklist promotes self-education in an appropriate work environment which is an added value [22]. Having a dedicated room for work, having an ergonomically correct workstation, knowledge of how to adjust a workstation, and increased satisfaction with indoor environment factors (microclimate, lighting, etc.) in the workspaces are associated with a lower chance of experiencing new health issues, e.g., decrease the incidence of musculoskeletal disorders [7,9]. Therefore, workplace risk assessment with identification of needed preventive measures combined with training of workers on ergonomics and encouragement to implement the needed measures are essential for telework.

### 1.5. Risk Assessment of Teleworkplaces in Latvia

When looking at the situation concerning workplace risk assessment in the case of telework in Latvia, the results of the project “Life with COVID-19: Evaluation of overcoming the coronavirus crisis in Latvia and recommendations for societal resilience in the future” (agreement No: VPP-COVID-2020/1-0013) show that only 26.4% of teleworkers in a web survey have reported that his/her employer has found out the teleworking conditions (the term “found out” was used to avoid a complicated legal term “workplace risk assessment”) [23]. Even more surprisingly, 42.6% of teleworkers believe that workplace risk assessment is not needed and should not be implemented. However, this group of respondents is not homogenous—it includes teleworkers who already had an ergonomic home office before the COVID-19 pandemic, who believe that workplaces located at worker homes are not the responsibility of the employer, and others [23]. The same study also analyzed health effects mentioned by teleworkers in association with workplace risk assessment—workers who report lack of risk assessment also report more health-related complaints, e.g., pain lasting longer than three days (26.9% of respondents with workplace risk assessment versus 73.1% of respondents without workplace risk assessment), sore eyes (24.4% versus 75.6%) and anxiety (21.8% versus 78.2%) [23]. The results of the same survey show that the odds of several analyzed self-reported health effects were significantly increased. For example, among the respondents who reported no workplace risk assessment, the odds of anxiety increased 2.83 times (95% CI 1.50–5.35), of sore eyes—2.81 times (95% CI 1.50–5.26), for pain longer than three days—1.82 (95% CI 0.95–3.51). In general, this study shows that workplace risk assessment provided by the employer can reduce the percentage of workers who have complaints related to sore eyes, anxiety, and pain longer than three days [24].

Telework during the COVID-19 pandemic was a huge challenge for employers to fulfill their legal occupational safety and health obligations, including ensuring healthy and safe working conditions and assessing workplace risk factors for workplaces located in the homes of their workers. This article focuses on the identification of methods used by employers for workplace risk assessment, identification of barriers and challenges for carrying out this legal obligation, as well as on the future trends in management of occupational safety and health at the company level.

## 2. Materials and Methods

Focus group discussions of two target groups (employers and occupational safety and health experts) were used as a qualitative research method.

### 2.1. Study Design and Recruitment of the Participants

To obtain data on measures taken by employers at the company level to arrange healthy and safe teleworking conditions and to support workers in this process, in total 10 focus group discussions were organized. Focus group participants were recruited voluntarily through public announcements, social media (Facebook and Twitter) posts, local employers’ non-governmental organizations, personal contact networks, and national labor inspectorate.

In total, 65 employers from 65 companies of different sizes and regions participated in 8 focus group discussions. The number of participants varied between 5 and 11 per group. The allocation of the employers to the relevant focus group was based on the size of the company (number of the workers in the company) and the region where the main premises of company are located. If the employer was representing a company with less than 100 workers, he/she was allocated to the focus group of small and medium-sized organizations. If the applicant was from the companies with 100 or more workers, he/she was allocated to the focus group of large organizations.

Two separate focus group discussions were organized for occupational safety and health experts with 12 participants in each group, representing 24 other companies. Experts providing external occupational safety and health services participated in one discussion, but persons working as internal occupational safety and health experts in companies were invited to another one. During the recruitment process of the occupational safety and health expert focus group participants, the information related to the employment was requested from the applicants to allocate them to the relevant group or exclude them from the participation in the survey. More information on the recruitment process and the focus group participants has already been published [25].

We obtained verbal consent from all participants—before starting of the focus group discussions, the moderator explained the purpose of the study and allowed the participants to ask questions. No monetary or other compensation was offered to the participants.

### 2.2. Procedure of the Focus Group Discussion

A standardized procedure was used to manage all focus group discussions. New legal requirements on the mitigation of the spreading of the SARS-CoV-2 virus were approved by the Latvian government after the beginning of the first focus group discussion. Therefore, some participants were interviewed on-site, others using online platforms Zoom and MS Teams. Experienced moderators (author I.V., technical assistants I.A., S.R.) led the discussions according to written guidelines which consisted of logically proceeding questions. All three moderators were supported by a note-taker and technical support person. Persons familiar with occupational safety and health but not belonging to the research team were involved in testing and validation of the questions.

The organization of telework was covered during the final part of the discussions. According to the developed guidelines of the focus group discussions, participants were invited to explain their personal teleworking experience and their experience in the company they represent. Spontaneous answers on teleworking experience were followed by open-ended questions on the organization of telework and legal aspects, risk assessment for teleworking workplaces, preventive measures provided by the employer (e.g., technical support with computers, IT consulting, chair, desk, etc.). For this article, we used only the following questions from the guidelines: “Did you check the working conditions of the teleworkers (did you provide workplace risk assessment)? How did you do it (did you use any tools; did you involve occupational safety and health expert of your company)? What kind of support did you provide for your workers that they work in physically and emotionally safe and healthy environment? Did the employer compensate costs arising from telework (e.g., for internet, electricity)?”. A shared PowerPoint presentation was used to share the relevant questions with online participants.

Records from all the focus group discussions were made after receiving oral permission from the participants. These recordings were later used for transcribing and matching information correctly. Recordings were uploaded, kept on the internal protected server of the Rīga Stradiņš University, taking into account the data protection rules.

Focus group discussions lasted between 115 to 152 min each, approximately 25 min per focus group were used to discuss various aspects of telework.

### 2.3. Data Analysis

Anonymized transcripts were drafted. During this process a researcher not involved in the organization of focus group discussions and the analytical process (L.A.) carefully de-identified participants. Further, conventional content analysis was conducted by two independent and experienced researchers. Both of these experts have backgrounds in occupational medicine and more than 20 years of practical experience in occupational safety and health (L.M., I.V.). This process was assisted by another researcher—a public health specialist, also working in the field of occupational safety and health (L.P.). Both researchers (L.M., I.V.) together read the anonymized transcripts and suggested the first possible categories and subcategories. No theoretical considerations were used to identify them. For our research, we decided to use building of the categories directly on the data from the focus groups. We believe that this method is more relevant to our research question.

The analysis of all of the focus group transcripts was performed step-by-step. Initially, two researchers independently did the coding of the transcript of the discussions of the external occupational safety and health experts. Then, during the meeting, the codes were compared, and the agreement on the codes to be used for the analysis of all other transcripts was reached. More subcategories were created during the further analytical process. The final set of the categories and subcategories to be presented as the results was created by collapsing and merging the initial ones. At the final stage, the assisting researcher (L.P.) reviewed all of the transcripts to verify the findings and to draft the table of the results.

The most relevant quotes were marked at all stages of the analysis of transcripts. After that, all researchers met to select examples that best explain the different answers to the questions and demonstrate various opinions and experiences. Although the selected quotes were anonymous, for quotes of the employers, we decided to provide the reference to the size and the region of the represented company (Riga and suburbs of Riga represent the capital of the country and the surrounding territory; Kurzeme, Zemgale, Latgale, Vidzeme are the geographical regions where the regional focus groups were organized). For quotes of occupational safety and health experts, we decided to provide only information on whether the person works as an internal occupational safety and health expert (“internal”) or provides occupational safety and health services (“external”). In the section of results, only one or two supporting quotes are provided, other relevant quotes are given in the Appendix A (Table A2).

Although the transcripts of the focus group discussions covered also topics related to preventive measures taken by the employers to improve the working conditions in home offices (e.g., in cases workers required an office chair, wrist support, a footrest, a special mouse, they were encouraged to take these items with them if they were working from homes), we decided to keep the focus of this article on workplace risk assessment and training of teleworkers, therefore other our findings are not covered in the section of the results.

During the analyses of transcripts, one of the informative materials on telework was identified to be repeated several times [26]. Since different participants referred to this material using different keywords, we decided to use the term “material developed by the public authorities” in all cases. In some cases, the used keyword was wrong which can be explained by the collaboration between different institutions. For example, although this material was prepared by the Institute of Occupational Safety and Environmental Health of Rīga Stradiņš University, during the first wave of the pandemic it was promoted on the social media accounts of the State Labour Inspectorate, therefore some of the focus group participants referred to it as the material of the State Labour Inspectorate.

## 3. Results

Six main themes of discussions were revealed: (1) methods used to identify/assess working conditions in home offices, (2) supportive factors for workplace risk assessment, (3) reasons for not providing risk assessment during the first wave of the COVID-19 pandemic, (4) other methods to improve working conditions in home offices, (5) involvement of workers, and (6) compensation of costs to improve working conditions (see Table 1).

When asked about workplace risk assessment for telework, most of the focus group participants (*n* = 64; 71.9%) acknowledged that working conditions of the teleworkers were not checked:

*“No, we were just thinking [about workplace risk assessment], but we did not provide one”*

*An internal occupational safety and health expert*

Discussions on teleworking conditions highlighted several reasons for not assessing workplace hazards. One of the main reasons was related to the fact that focus group participants (*n* = 4; 6.3% of the focus group participants not providing workplace risk assessment for teleworkplaces) believed that in cases when the home office is not the only workplace (the worker also has a workplace on the premises of the employer), then it is enough to provide risk assessment for the workplaces located in the premises of the employer. In addition, some other participants (*n* = 2; 3.1% of the focus group participants not providing workplace risk assessment for teleworkplaces) hoped that telework was used temporarily during the first wave of COVID-19, and therefore there was no need to do it:

*“We did not have a risk assessment for distant workplaces. Telework was relative—we worked 50:50 [50% of the time in the premises of the employer, 50% of the time—at home]”*

*An internal occupational safety and health expert*

*“I think, if the second wave [of the COVID-19 pandemic] will come, we will more focus on workplace risk factors at home”*

*A large company from Riga, suburbs of Riga*

Lack of understanding on the possible process of doing a workplace risk assessment in case of telework was even mentioned by qualified occupational safety and health experts who have postgraduate education in occupational safety and health (*n* = 6; 25% of all qualified occupational safety and health experts who participated in the focus group discussions; 43.9% of those qualified occupational safety and health experts who had not provided workplace risk assessment). This stresses the need to focus on further training activities of occupational safety and health experts to act in non-standard situations and to deal with new and emerging risks:

*“Everything is so unclear…Even now [four months after the end of the first emergency state]…”*

*An external occupational safety and health expert*

In addition, some focus group participants (*n* = 6; 9.4% of those focus group participants not providing workplace risk assessment for teleworkplaces) pointed out the problems related to legal acts covering occupational safety and health for telework (new legal requirements related to telework were accepted already before the first emergency state; however, they came in force only after the end of it). Some of the participants explained that they were not able to understand the legal requirements, they were too bureaucratical and therefore the workplace risk assessment should be left to be done by the teleworkers themselves:

*“As for me, it is still unclear how these questions [on workplace risk assessment in telework] are regulated in our country. From July, telework is covered by occupational health and safety legal acts, but it is not clear, how companies can perform it and what exactly should be done”*

*A large company from Kurzeme/Zemgale*

*“…and also—workplace risk assessment should be left to be done by the worker himself and not by the company”*

*A large company from Latgale*

Among the reasons why workplace risk assessment was not performed, privacy issues, as well as lack of interest of workers to provide any information about their homes, were mentioned by some of the focus group participants (*n* = 5; 7.8% of those focus group participants not providing workplace risk assessment for teleworkplaces):

*“I can’t step into the privacy and request to send a photo; I can’t visit his kitchen or wherever he works. It is a very general question”*

*A small/medium company from Riga, suburbs of Riga*

When looking for the factors which have promoted workplace risk assessment for teleworking workplaces, teleworking experience before the COVID-19 pandemic was stressed by several focus group participants (*n* = 8; 32% of those focus group participants who had provided workplace risk assessment for teleworkplaces). A few of the participants specified that they have already done such workplace risk assessment for at least several workplaces, and therefore it was easier for them to manage occupational safety and health in forced telework during the COVID-19 pandemic:

*“We started to think on telework, and digitalization in general, already some time ago. All workstations were equipped with laptops, big monitors, so, transition to telework was rather smooth…Actually, we were more ready”*

*A large company from Riga, suburbs of Riga*

When asked about the methods for doing a risk assessment of teleworkplaces, despite COVID-19 epidemiological restrictions, a few focus group participants (*n* = 3; 12% of those focus group participants who had provided workplace risk assessment for teleworkplaces) mentioned the use of the traditional approach—visiting of the workplace (in this case, workers’ homes) and inspection of the workstation:

*“In one IT company, where everyone could telework, a special person was employed which visited each worker at home. This company had elaborated an internal checklist listing all requirements for the workplace. If something was missing, it was provided by the company, however, if there was not a special room for the home office, the worker was not allowed to telework and he had to work from the employer’s office. It was a good example—they provided computer, table, chair and so on”*

*An external occupational safety and health expert*

More often focus group participants mentioned several other methods used by the employers: self-addressed checklists filled in by the workers themselves (*n* = 14; 56% of those focus group participants who had provided workplace risk assessment for teleworkplaces), interviews of teleworkers (without seeing the work station) (*n* = 2; 8% of those focus group participants who had provided workplace risk assessment for teleworkplaces), and photos (and in rare cases also short videos) sent in by the workers (*n* = 6; 24% of those focus group participants who had provided workplace risk assessment for teleworkplaces):

*“We organized an employee survey…and it contained also questions that allowed them to assess their workplaces—e.g., do you have enough fresh air, do you have enough space, we asked for square meters…So, they answered “Yes” or “No”…”*

*An internal occupational safety and health expert*

*“I elaborated a checklist, I based it on that good material [developed by the public authorities]. I sent the checklist out, invited to fill it in, and if there were problems or some tiny things to be improved, then I or someone from the Human Resource department got in touch and discussed the situation. I am not sure if legally this is a risk assessment, but it was effective”*

*An external occupational safety and health expert*

*“Workers also sent in photos from their workplace or short videos with a request—can you tell what else I can do to improve my working conditions?”*

*A small/medium company from Riga, suburbs of Riga*

Half of the focus group participants who had used checklists (7 out of 14) mentioned that they have used checklists or based their checklist on the material prepared by public authorities, which stresses the value of advising and consulting activities provided by the state (e.g., the Ministry of Welfare, the State Labour Inspectorate, the Institute of Occupational Safety and Environmental Health):

*“We asked our employees about the working conditions and used the material [developed by the public authorities]”*

*A large company from Riga, suburbs of Riga*

In addition to the workplace risk assessment, several focus group participants mentioned training on office ergonomics which was adapted to the online form (*n* = 11; 12.5% of all focus group participants):

*“An essential thing is training, I made an e-learning course on how to better work from home, how to have a comfortable workplace…”*

*A small/medium company from Riga, suburbs of Riga*

Instead of an online training course, some of the focus group participants (*n* = 6; 6.7% of all focus group participants) mentioned sending out information on safe and healthy telework, e.g., guidelines, safety instruction, recommendations, infographic, etc.:

*“We immediately sent out information on how to arrange the workplace at home, how to manage these risks…in order not to have back pain after three months”*

*An internal occupational safety and health expert*

One of the focus group participants stressed the need for regular provision of information that can promote the creation of more ergonomic workplaces:

*“What I do—I call it the slow dropping. I add some more information every day—a very small droplet. Then they read, look at the pictures, start to think, but it is a long work”*

*A small/medium company from Riga, suburbs of Riga*

Some of the focus group participants (*n* = 6; 6.7% of all focus group participants) pointed out that their workers were very much interested in getting advice on how to improve their working conditions in home offices:

*“Workers were very active in giving answers when we got in touch with them, they took items [they needed from offices] to their homes…and everything worked”*

*A large company from Riga, suburbs of Riga*

Several focus group participants (*n* = 6; 6.7% of all focus group participants) mentioned the need for increased responsibility of workers who telework:

*“I have to say that I think that it [workplace risk assessment for telework] is very much linked with activation of personal responsibility because workers often think they don’t need anything from the employer, but others clearly know what exactly they need”*

*A small/medium company from Riga, suburbs of Riga*

Another aspect discussed in the focus groups was the compensation of costs arising from telework, including the topic on who should pay for the improvement of ergonomics in teleworkplaces. Only some of the participants (*n* = 4, 4.5% of all focus group participants) mentioned that their employer had compensated some costs, mainly related to internet and electricity; however, more participants (*n* = 9; 10.1% of all focus group participants) reported that their employer allowed taking the needed equipment from the offices to workers’ homes (e.g., office chairs, office desks with adjustable height, computer mice, ergonomic keyboards, lamps, etc.):

*“Some workers were compensated costs if they needed, but all of them could take everything from office to their homes…”*

*A small/medium company from Riga, suburbs of Riga*

## 4. Discussion

Although traditionally health and safety related workplace hazards are identified and managed through workplace risk assessment [15], the emergency situation related to the spread of SARS-CoV-2 has shown that neither employers nor occupational safety and health experts were able to respond to the rapidly changing working life, including management of telework. During the first wave of the COVID-19 pandemic, workplace risk assessment for home offices was provided in rare cases. When analyzing the dominating reasons for the low coverage of teleworkers with workplace risk assessment, we managed to identify two main groups of reasons. The first group of reasons includes aspects which have resulted from the COVID-19 pandemic, e.g., lack of time (the employers had to focus on other aspects of business, such as keeping business or adjusting it to the rules of the pandemic) or delayed deliveries (as it was impossible to implement identified preventive measures, there was no sense in doing risk assessment), or GDPR and privacy issues (workers refusing to provide information on their workplaces located at their homes).

The other groups of reasons summed up as excuses used by the employers and occupational safety and health experts to justify not doing workplace risk assessment including the “Let’s wait” strategy, comments explaining that the legal requirements are unclear/complicated/exaggerated, or mentioning that there is no need for doing workplace risk assessment if telework is part-time or done by a low proportion of workers. Lack of understanding of the possible process for implementation of workplace risk assessment in the case of telework was mentioned even by qualified occupational safety and health experts who have postgraduate education in occupational safety and health, and this raises questions if the existing training system is effective for preparing experts to act in non-standard situations and to deal with new and emerging risks.

When choosing the method for workplace risk assessment, it is essential not to overcomplicate the process [15]. However, the COVID-19 pandemic has shown the need of moving away from general workplace risk assessment (valid for more than one workplace) to a personalized assessment (valid for only one workplace). Office workplaces situated in the premises of the employer are more or less similar in the terms of available office furniture, computers, ventilation, etc. This is not applicable for the home offices which are arranged by teleworkers themselves and using the available furniture [7,9], therefore each situation should be assessed individually.

We have identified some authors pointing out the ergonomic principles to be used in home office arrangement: “Create a working space with a computer that is ergonomically friendly (a screen of adequate size) and with an appropriate desk. The more it resembles your workplace, the better. A second monitor would replace the need to print documents and it is important to find a space away from your bedroom to work.…Make sure your working space also has a comfortable chair, good illumination and ventilation and adequate accessories (such as a good internet connection and microphone and camera)…” [27]. However, from a legal point of view, this is part of the workplace risk assessment is to be done by the employer or an occupational safety and health expert designated by the employer. During the workplace risk assessment, persons performing it should walk around the workplace and look at what could reasonably be expected to cause harm [15]. This task was extremely complicated with the COVID-19 pandemic restrictions when workplace visits and observations of the working process were impossible due to different reasons, e.g., epidemiological restrictions, physical distancing, privacy issues, etc. In such situations, more advanced employers and occupational safety and health experts may wish to consider conducting a virtual site-check to ensure that the potential health and safety hazards are assessed and that steps are taken to mitigate them [19].

Our research has identified two main methods for identification and assessment of workplace risk assessment used by the employers: self-addressed checklists filled in by the workers themselves and photos (and in rare cases also short videos) sent in by the workers. Different non-scientific sources also describe similar approaches, e.g., (1) “Carry out a risk assessment of the home working environment including any workstation, for example, by asking the workers to complete a self-assessment questionnaire and take photographs then having a trained risk assessor evaluate the results” [28]; (2) “Employers may wish to ask employees to complete and submit a risk assessment (with photographs if appropriate) for their workstation if they are to work from home which can be virtually assessed by the human resource team who can provide assistance and guidance” [29]; (3) “To help you out, we have created a simple risk assessment template for you to download and help you assess your home workspace” [30].

When analyzing what kinds of checklists were used in Latvia, it is important to stress the work of the public authorities which had provided advice and informative materials. In general, at the beginning of the COVID-19 pandemic most employers and occupational safety and health experts felt confused about their obligations; however, when looking for sources of information, most of the focus group participants mentioned websites of the state authorities as well as the national working life portal www.stradavesels.lv (accessed on: 18 September 2021) [23].

When looking in detail at doing risk assessment by reviewing photos, it is not just sending in random photos from the workplace. Two photos for ergonomic assessment should be submitted for an experienced person (e.g., occupational safety and health expert) for evaluation—one from directly behind the person using the computer in the home office area and one from the side [11]. This would mean that before taking photos the worker should receive precise instructions [11]. Taking into account research showing that during telework workers have reduced access to ergonomics information and resources, in addition to the instructions on taking photos, employers should offer their workers also ergonomics and safety training or resources to change work habits and improve the physical home-based work environment [14,19]. Proper training in ergonomics has been found to be effective in teleworkers [7,31]. However, it is not just the traditional training on office ergonomics; the key messages must be specifically tailored to adapt their homes and lifestyles to telework [20]. Such combined activities involving workplace risk assessment and workers’ training seem to be more effective than just one isolated measure.

COVID-19 has caused a lot of questions for occupational safety and health experts on workplace risk assessment for home offices. Already before the COVID-19 pandemic, the home assessment had been used by other industries, e.g., in occupational therapy [32]. These home assessments in occupational therapy aim to identify hazards or environmental issues which may affect safety or function and to determine possible strategies to overcome these hazards or issues, including home modifications [33]. In principle, this process is the same as workplace risk assessment for home workplaces, only the person is different. In occupational therapy, the person for which the assessment is done is a person with serious health issues, but workplace risk assessment is done for a worker working from home. Research shows that occupational therapists who have conducted home assessments implemented twice as many recommendations as those undertaking only in-hospital consultations [33]. Transferring these findings to occupational safety and health, it would mean that visits to home offices for assessment of working conditions and identification of preventive measures should be encouraged. However, during the COVID-19 pandemic home visits were not possible due to epidemiologic restrictions. This suggests the transfer of other experience from occupational therapy—occupational therapists already before the COVID-19 pandemic had explored the use of video conferencing equipment, digital photographs, and digital report writing for home assessments [33]. In addition, research has shown that direct communications between a diabetes patient and a clinician via Skype appeared to help supported self-adjustment of insulin dosage [34]. According to our understanding, if online communication is helpful for self-adjustment of insulin dosage, it can also be helpful for workplace risk assessment, adjustment of an office chair or implementing any other ergonomic improvements.

Another aspect to be considered for transfer from occupational therapy to occupational safety and health is related to the fact that the introduction of video consultations also brings operational and cultural challenges, including the need to develop new ways of organizing work, to train and support both staff and clients in technology use [34]. That would be especially important in the work of external occupational safety and health service providers which might need to change the way they provide their consultations and expertise.

Not only knowledge but also concerns should be transferred from occupational therapy to occupational safety and health. In occupational therapy, concerns had been noted about whether a virtual visit would replace the traditional visit completely and this seemed to cause concern for professional practice [35]. This is also very applicable for occupational safety and health—if doing workplace risk assessment without a visit to the workplace is accepted for telework, then why is the same approach not used for any other workplaces? This raises questions about the future developments of the workplace risk assessment process and the influence of digital technologies on the work of occupational safety and health experts, especially external service providers who are not in the same company on an everyday basis.

Our research shows that, although the main reason for doing workplace risk assessment is a determination of preventive measures to be taken to ensure a healthy and safe working environment, only a few employers and occupational safety and health experts provided feedback with suggested improvements after receiving filled-in questionnaires or photos from home offices. Giving feedback to the teleworkers on effective practices to improve working conditions by occupational health physicians and nurses has been proven to be effective, therefore feedback from experienced occupational safety and health experts should also be encouraged, especially if the recommendations are viable and inexpensive [11,22]. However, workplaces that lack the above-mentioned specialists may not be able to respond effectively to achieve the expected health effects [22]. Another aspect connected to the improvement of home office ergonomics is the costs related to the preventive measures. Our research shows that the COVID-19 pandemic has required the employers to be creative. Instead of buying new equipment, most employers offered their workers to take the needed equipment home from the office. However, this is a temporary solution because if the workers will partly return to offices, they will need those office desks, chairs, monitors in both workplaces—in the office of the employer and in the home office. In such situations, we have to take into account the requirements of the Council Directive 89/391/EEC of 12 June 1989 on the introduction of measures to encourage improvements in the safety and health of workers at work which states that “measures related to safety, hygiene and health at work may in no circumstances involve the workers in financial cost” [3].

The main principles of conducting a good workplace risk assessment cover also the collaboration with workers and/or their representatives. It is essential to work together [15]. It seems that the involvement and the role of the teleworkers in the workplace risk assessment have rapidly increased during the COVID-19 pandemic as there is no way for employers and occupational safety and health experts to provide a qualitative and comprehensive risk assessment with tailored preventive measures without knowing exactly what the working conditions in home offices are. This has been recognized also by some governments, e.g., Spain established an exceptional temporary legal measure allowing workplace risk assessments for home offices to be carried out by the worker based on a provided checklist [1].

The involvement of teleworkers in workplace risk assessment is also related to privacy issues. When the teleworkplace is in the home of the worker, any request to submit information can be perceived as surveillance of workers and the whole particular household done by the employer [36]. Fears of such surveillance may set potential teleworkers, employers of teleworkers, and trade unions against this mode of employment, regardless of whether such fears are justified [36].

The decisions of the Latvian government on legal requirements on mitigation of spreading of the SARS-CoV-2 virus approved after the beginning of the first focus group discussion have resulted in one of the limitations of our study—some of the participants expressed their opinion in person, and others using online tools (for details see [25]). Although experienced moderators used different methods to overcome this problem (e.g., questions were addressed to the focus participants individually), the equality of every single participant might have been affected.

We also faced problems while trying to recruit focus group participants from companies that are not known as good practice examples for management of occupational safety and health. Most of the focus group participants represent companies that can present good examples in most aspects of health, safety and well-being of their workers, and also the management of new risks related to the COVID-19 pandemic. These companies are also very keen to share their activities. Therefore, we assume that the focus group participants over represent companies with well-established occupational safety and health management systems and the situation in other companies could be significantly worse.

## 5. Conclusions

If the findings from our study are combined with the results of the web survey of the Latvian teleworkers (only 26.4% of teleworkers have reported that his/her employer had provided workplace risk assessment during the first wave of the COVID-19 pandemic) [23], we can assume that at least part of the employers in Latvia in the situation of COVID-19 pandemic have not sufficiently implemented their legal obligations related to workplace risk assessment for home offices which might result in increased numbers of physical and mental health problems of teleworkers in the future. Work from home has shown how working conditions can differ for the same type of work (office work), therefore, the promotion of personalized workplace risk assessment should be encouraged.

Even if virtual workplace visits using photos and videos are not the traditional way the workplace risk assessment should be done, we believe it is effective. Workers who report that their employers were aware of their working conditions report fewer health effects and that means it has reached the main aim specified in the Framework Directive on occupational safety and health. This might lead to further discussions—if a virtual workplace risk assessment is effective for home offices, why not use it also for other workplaces?

Workplace risk assessment for teleworking workplaces might change the level and role of worker participation in workplace risk assessment. We believe that most likely also after returning to the premises of the employer these workers will be more interested in the improvement of their workplaces, and this can promote their active involvement in the management of health and safety at work in general. However, it can also lead to the situation that companies performing best in occupational safety and health become even better, but companies with a low level of occupational safety and health management do not improve much.

## Figures and Tables

**Table 1:** Themes identified during research analysis (*n* = number of persons for which theme was detected).

Main Category	Subcategory	Employers (*n* = 65)	OSH * Experts (*n* = 24)	In Total (*n* = 89)
Methods used to identify/assess working conditions in home offices	A questionnaire/checklist was sent out to be filled in by the worker himself/herself	*n* = 8	*n* = 6	*n* = 14
	Feedback with suggested improvements was provided by the employer/occupational safety and health expert (after receiving information from workers)	*n* = 5	*n* = 3	*n* = 8
	Material elaborated by the public authorities was used	*n* = 4	*n* = 3	*n* = 7
	Workers sent in a photo or a video from the home office	*n* = 5	*n* = 1	*n* = 6
	Personal visits to home offices were available	*n* = 2	*n* = 1	*n* = 3
	Unstructured telephone interviews with workers were held	-	*n* = 2	*n* = 2
Supportive factors for workplace risk assessment	Teleworking experience in the company before the COVID-19 pandemic	*n* = 6	*n* = 2	*n* = 8
	Good occupational safety and health performance (including well-trained workers in occupational safety and health) in the company before the COVID-19 pandemic	*n* = 3	*n* = 2	*n* = 5
Reasons for not doing workplace risk assessment during the 1st wave of COVID-19 pandemic	No telework, part-time telework, low percentage of teleworkers	*n* = 7	*n* = 4	*n* = 11
	Unclear/complicated/overexaggerated legal requirement	*n* = 3	*n* = 3	*n* = 6
	GDPR, privacy issues	*n* = 5	-	*n* = 5
	“Let’s wait” strategy	*n* = 3	*n* = 1	*n* = 4
	Lack of time	*n* = 2	-	*n* = 2
	Risk assessment was already done before COVID-19	*n* = 2	-	*n* = 2
	Delayed deliveries (impossible to implement identified preventive measures)	*n* = 1	-	*n* = 1
Other methods to improve working conditions in home offices	Online training for workers on arrangement of home office	*n* = 7	*n* = 4	*n* = 11
	Written teleworking guidelines/instructions with ideas to improve teleworking conditions	*n* = 3	*n* = 3	*n* = 6
Involvement of workers	Importance of personal responsibility of the worker	*n* = 6	-	*n* = 6
	Invitation of workers to request needed improvements	*n* = 3	*n* = 3	*n* = 6
Compensation of costs to improve working conditions	Compensation of costs related to internet and electricity	*n* = 2	*n* = 2	*n* = 4
	Permission to take equipment from office to home office (as alternative to compensation of costs)	*n* = 7	*n* = 2	*n* = 9

* Occupational safety and health.

## Data Availability

Data sharing of focus group discussions is not applicable as the data consists of focus group transcripts, which for reasons of confidentiality, cannot be shared.

## Appendix A

**Table A1:** Percentage of teleworkers (%) in Latvia by age groups, 2020 and 2021.

Period	Status of Epidemiologic Situation	In Total (15–74)	Age Groups			
			15–34	35–44	45–54	55–74
2nd quarter of 2020	1st emergency state (12 March and 9 June 2020)	18.3	12.4	21.7	19.7	20.1
3rd quarter of 2020	-	8.9	8.4	12.6	8.6	6.3
4th quarter of 2020	2nd emergency state (9 November 2020 and 6 April 2021)	18.0	20.0	21.9	17.9	11.9
1st quarter of 2021		22.6	27.0	25.1	22.0	15.8

Source: Official statistics portal. Official statistics of Latvia [37].

**Table A2:** The most relevant quotes to support the categories.

Supporting Quotes	Reference to the Author of the Quote
1. Working conditions of the teleworkers were not checked	
“But those workers who have chosen telework, no, their working conditions were not checked”	A large company from Latgale
“Well, it seems, we are an example of how not to do anything related to this topic. We did not have enough time for all this”	A large company from Riga, suburbs of Riga
No, we did not keep track [if the worker has adequate working conditions]”	A large company from Vidzeme
“No, we were just thinking [about workplace risk assessment], but we did not provide one”	An internal occupational safety and health expert
“Currently [four months after the end of the first emergency state due to COVID-19] risks for telework are not assessed. We will do it, but we started with providing laptops, signed agreements between the employer and worker that the workers must be available via phone and email”	A large company from Riga, suburbs of Riga
“About the workplaces…I will be honest, nobody was specifically checking these working conditions, but everybody tried to adjust. I was teleworking and, yes, I used an ironing desk and was sitting on a sofa”	A large company from Riga, suburbs of Riga
2. Reasons for not providing workplace risk assessment	
“We did not have a risk assessment for distant workplaces. Telework was relative—we worked 50:50 [50% of the time in the premises of the employer, 50% of the time—at home]”	An internal occupational safety and health expert
“I think, if the second wave [of the COVID-19 pandemic] will come, we will more focus on workplace risk factors at home”	A large company from Riga, suburbs of Riga
Lack of information, confusion, unclear legal acts	
“With workplace risk assessments… Yes, I felt very confused—do I really have to visit everyone at home?”	An external occupational safety and health expert
“Anyway, this is the big question—how to organize risk assessment process?”	An external occupational safety and health expert
“Everything is so unclear…Even now [four months after the end of the first emergency state]…”	An external occupational safety and health expert
“What should we do? Do we need safety instructions, how should we assess risks—I received so many questions on all these topics”	An external occupational safety and health expert
“As for me, it is still unclear how these questions [on workplace risk assessment in telework] are regulated in our country. From July, telework is covered by occupational health and safety legal acts, but it is not clear, how companies can perform it and what exactly should be done”	A large company from Kurzeme/Zemgale
“There is a too high bureaucracy in the Labour Protection Law, especially concerning workplace risk assessment for distant workplaces”	A large company from Latgale
“…and also—workplace risk assessment should be left to be done by the worker himself and not by the company”	A large company from Latgale
“The employer cannot be responsible for the working environment in the home of the workers”	A large company from Latgale
“Usually, everything is solved after the first court decision. By now, there is no such decision...”	A large company from Kurzeme/Zemgale
Privacy issues	
“Risk assessment for work from home—we did not do it. Firstly, this very much related to GDPR”	A large company from Riga, suburbs of Riga
“I can’t step into the privacy and request to send a photo; I can’t visit his kitchen or wherever he works. It is a very general question”	A small/medium company from Riga, suburbs of Riga
“About the risk assessment—the current situation is so that checklists, personalized surveys are effective now, but workers do not want to send these photos or videos. Some of them are interested, but most of them want to keep their personal space as a private one”	A small/medium company from Riga, suburbs of Riga
3. Factors promoting workplace risk assessment	
“I had already started to do something [on workplace risk assessment already before COVID-19], but March [of 2020] promoted this rapidly”	An internal occupational safety and health expert
“We started to think on telework, and digitalization in general, already some time ago. All workstations were equipped with laptops, big monitors, so, transition to telework was rather smooth…Actually, we were more ready”	A large company from Riga, suburbs of Riga
4. Methods of workplace risk assessment	
Surveys and checklists	
“We organized an employee survey…and it contained also questions that allowed them to assess their workplaces—e.g., do you have enough fresh air, do you have enough space, we asked for square meters…So, they answered “Yes” or “No”…”	An internal occupational safety and health expert
“About workplace risk assessment—no, I did not visit anybody at home, but I had a special checklist based on those pictures [of the material developed by the public authorities]. Workers filled in, and then we agreed what else they should do in their home offices”	An external occupational safety and health expert
“I elaborated a checklist, I based it on that good material [developed by the public authorities]. I sent the checklist out, invited to fill it in, and if there were problems or some tiny things to be improved, then I or someone from the Human Resource department got in touch and discussed the situation. I am not sure if legally this is a risk assessment, but it was effective”	An external occupational safety and health expert
“...and then we started with workplace risk assessment. Workers had distance training, how this self-assessment for teleworking workplaces should be done. We worked individually with all workers on this workplace risk assessment and we assisted if something was needed”	A large company from Kurzeme/Zemgale
“In that checklist, we have questions which were assessed by our legal department [due to the privacy issues]. Then we have online training, where we discuss these questions one by one. And then we sign the agreement with workers explaining that it is his/her responsibility to work in that particular workplace. Basically, with the self-assessment, we want to be sure that he/she has at least one healthy and safe workplace”	A large company from Kurzeme/Zemgale
“We made kind of survey with the checklist, where the workers themselves did risk assessment...”	A small/medium company from Kurzeme/Zemgale
Photos, videos, phone calls	
“With risk assessment, it was rather similar—we allowed to take chairs home, but of course we asked to send photos afterward”	A small/medium company from Riga, suburbs of Riga
“We have provided all office workers with recommendations on arrangement of the home office, we sent them also pictures from that popular material [prepared by the state authorities]”	A small/medium company from Riga, suburbs of Riga
“[Workers] informed us with photos, they showed how they manage [to work] from home”	A small/medium company from Riga, suburbs of Riga
“Workers also sent in photos from their workplace or short videos with a request—can you tell what else I can do to improve my working conditions?”	A small/medium company from Riga, suburbs of Riga
“We assessed risks for telework in general, but it is difficult to do it at the individual level. You can assess them only when the worker sends you a photo or you can visit them, but it is only voluntarily”	A small/medium company from Riga, suburbs of Riga
“…. and we have recommendations how such [distant] workplaces should be arranged. We have elaborated a new approach which we tested in some companies—we asked questions either by emails or by phone, but did not inspect any workplace. It was more by phone or questionnaires”	An external occupational safety and health expert
“I assessed risk for telework by phone”	An external occupational safety and health expert
Materials prepared by the public authorities	
“We asked our employees about the working conditions and used the material [developed by the public authorities]”	A large company from Riga, suburbs of Riga
“From time to time, I reminded the workers on the existence of the checklist [prepared by the public authorities] as the illustration of the home office was very obvious and precise. It was very useful for work with employees!”	A large company from Riga, suburbs of Riga
“We sent out that picture [prepared by the public authorities]”	An internal occupational safety and health expert
5. Training on office ergonomics	
Online training	
“Internal e-training…covered topics related to ergonomic workplaces, how to do exercises, how to reduce stress level and so on”	A large company from Riga, suburbs of Riga
“An essential thing is training, I made an e-learning course on how to better work from home, how to have a comfortable workplace…”	A small/medium company from Riga, suburbs of Riga
“I organized online training for everyone. I prepared a presentation with tricks and tips—how can you improve working conditions in your home office, e.g., by using ironing desk, etc.”	A small/medium company from Riga, suburbs of Riga
Sending out information on safe and healthy telework	
“We immediately sent out information on how to arrange the workplace at home, how to manage these risks…in order not to have back pain after three months”	An internal occupational safety and health expert
“I elaborated guidelines for my companies, I sent them out, and of course, I wrote the occupational health and safety instruction for distance work. It is so popular!”	An external occupational safety and health expert
“The employer supported by sending out information on [the teleworking conditions]”	A small/medium company from Vidzeme
“What I do—I call it the slow dropping. I every day add some more information—a very small droplet. Then they read, look at the pictures, start to think, but it is a long work”	A small/medium company from Riga, suburbs of Riga
6. Worker involvement	
“We had colleagues which asked us [occupational safety and health experts] to check the workplaces—they sent in photos and we provided advice on what to do and how to improve the workplace”	An internal occupational safety and health expert
“Workers were very active in giving answers when we got in touch with them, they took items [they needed from offices] to their homes…and everything worked”	A large company from Riga, suburbs of Riga
“I want to support [another participant of the focus group discussions] on the personal responsibility. It is a hard and long task to achieve that the workers understand the consequences. It can’t be achieved in one day”	A small/medium company from Riga, suburbs of Riga
“I have to say that I think that it [workplace risk assessment for telework] is very much linked with activation of personal responsibility because workers often think they don’t need anything from the employer, but others clearly know what exactly they need”	A small/medium company from Riga, suburbs of Riga
7. Compensation of costs arising from telework	
“Some workers were compensated costs if they needed, but all of them could take everything from office to their homes, e.g., chairs from offices, adjustable office desks, mice, ergonomic keyboards, lamps…everything what they need”	A small/medium company from Riga, suburbs of Riga
“[The employer] offered to take home things what the workers need. Other costs were not compensated”	A large company from Latgale
“We paid for the internet which was used through hot-spots on mobile phones. There were also workers who brought home monitors, office chairs. This was the way to create an ergonomic workplace”	A large company from Riga, suburbs of Riga
“Workers could take the equipment from the office”	A small company from Latgale
